# Comparison of B cells' immune response induced by PEDV virulent and attenuated strains

**DOI:** 10.3389/fmicb.2024.1344344

**Published:** 2024-03-22

**Authors:** Chen Yuan, Xue Zhao, Yawen Feng, Ligong Chen, Yidan Lin, Tanqing Li, Qinye Song

**Affiliations:** ^1^College of Veterinary Medicine, Hebei Agricultural University, Baoding, China; ^2^Veterinary Biological Technology Innovation Center of Hebei Province, Baoding, China; ^3^Hebei Provincial Institute of Veterinary Drug Control, Shijiazhuang, China

**Keywords:** PEDV, B cells, surface molecules, cytokines, antibody

## Abstract

Porcine epidemic diarrhea virus (PEDV) is an acute, highly contagious enterovirus that infects pigs of all ages. The B cells are important for antigen presentation, antibody production, and cytokine secretion to resist infection. However, the role of B cells in PEDV infection remains unclear. In this study, the effects of PEDV virulent (QY2016) and attenuated strains (CV777) on B cells sorted from neonatal piglets, nursery piglets, and gilts were investigated. The results showed that PEDV-QY2016 and PEDV-CV777 could significantly increase the expression of CD54 and CD27 in B cells from neonatal piglets. The percentages of CD80, MHC II, and IgM expressed on neonatal piglet B cells infected with PEDV-QY2016 were significantly lower than those expressed on the B cells infected with PEDV-CV777. Both PEDV-QY2016 and PEDV-CV777 could stimulate IFN-α and GM-CSF secretions in neonatal piglet B cells; IL-1, IFN-α, and IL-4 secretion in nursery piglet B cells; and IL-1, TGF-β secretion, and GM-CSF in gilt B cells. Furthermore, both PEDV-QY2016 and PEDV-CV777 could induce the secretion of IgA, IgM, and IgG in nursery piglet B cells but could not induce the secretion of IgA, IgM, and IgG in neonatal piglet B cells. The secretion of IgA, IgM, and IgG was significantly higher by the PEDV-CV777 strains infected B cells than those by the PEDV-QY2016 strains infected gilt B cells. In conclusion, the surface molecule expression, cytokine secretion, and antibody production of B cells induced by PEDV are closely related to the ages of pigs and the virulence of the PEDV strain.

## Introduction

Porcine epidemic diarrhea virus (PEDV) is an enveloped, single-stranded, positive-sense RNA virus that belongs to the family *Coronaviridae* (Duarte et al., 1993). Although the virus could infect all ages of pigs via fecal-oral transmission, mortality in neonatal piglets is particularly high and could reach up to 100%. Similar to other porcine enteric coronaviruses, PEDV infection causes vomiting, acute diarrhea, decreased appetite, and dehydration. Following a large outbreak around 2010, PEDV has emerged as an imminent threat in the swine industry worldwide (Jung and Saif, 2015; Wang et al., 2016; Zhang et al., 2023). Recently, a few strategies have been employed to control PEDV outbreaks (Langel et al., 2016; Jung et al., 2020). However, PEDV still occurs frequently due to rapid virus mutation, which has led to tremendous economic losses in the swine industry worldwide.

The complex interactions between PEDV and the host immune system drive the process of PEDV infection, in which the adaptive immune system plays an important role in inducing a PEDV-specific immune response. The initiation of the adaptive immune response depends on antigen-presenting cells (Brodsky and Guagliardi, 1991). The activation and proliferation of these antigen-presenting cells are regulated by the interaction of specific antigens with their receptors. The antigen-presenting cells could capture and present the antigens to T cells or B cells. Cellular immunity mediated by T cells is important for defense against intracellular microbes. Humoral immunity mediated by antibodies that are secreted by B cells is mainly a defense against extracellular microbes (Lam et al., 2020).

At present, the relationship between PEDV infection and T cells has been described, which is mainly characterized by poor effector cytotoxic activity (Yuan et al., 2021). The knowledge of the role of B cells has been primarily focused on antibody secretion during PEDV infection (Huang et al., 2019). B cells that recognize antigens proliferate and differentiate into plasma cells that secrete different classes of antibodies. These antibodies can both neutralize and clear viral particles before the virus entry into the target cell (de Arriba et al., 2002; Dang et al., 2014). In fact, as an important part of the adaptive immune response, a variety of immune functions of B cells, such as antibody secretion, antigen presentation, and immune regulation, jointly mediate PEDV infection (Langel et al., 2016). However, the role of B cells in PEDV infection is often overlooked, and questions regarding the mechanism of B cell immune response induced by PEDV remain unclear.

In this study, we aimed to provide information on the immune mechanisms that occur after PEDV infection. We compared the effects of PEDV infection on phenotypic changes, cytokine secretion, and antibody production of B cells from pigs of different ages. The similarities and differences of B cells' immune responses induced by PEDV virulent and attenuated strains were compared. This study lays a foundation for an in-depth exploration of the infection and immune mechanism of PEDV and guides the research and development of more effective vaccines that induce broadly protective and long-lasting antiviral immunity.

## Materials and methods

### Virus

PEDV-QY2016 strains (Gen Bank Accession No. MH244927) and PEDV-CV777 strains (Gen Bank Accession No. KT323979) were stored in the Animal Infectious Disease Laboratory of the College of Veterinary Medicine, Hebei Agricultural University, Baoding, China.

### Reagents and antibodies

Porcine peripheral blood lymphocyte separation kit and red blood cell lysing buffer were purchased from Solarbio Science & Technology (Beijing) Co., Ltd. Enzyme-Linked Immunosorbent Assay (ELISA) kits were purchased from Dongge Biological (Beijing) Co., Ltd. Mouse Anti-Porcine CD21 IgG was purchased from Southern Biotech (Alabama). Anti-FITC MicroBeads and MiniMACS Starting kits were purchased from Miltenyi Biotec (Bergisch-Gladbach). Anti-CD54/ICAM1 antibody was purchased from Arigo (Taiwan). Mouse IgG1 kappa Isotype Control was purchased from Invitrogen (Waltham). Goat anti-Pig IgM was purchased from Bio-Rad (California). CD80 (B7-1) Monoclonal Antibody was purchased from eBioscience (California). Mouse anti-Pig-SLA Class II DR and mouse anti-Mouse MHC Class I were purchased from AbD Serotec (Kidlington).

### Generation of porcine blood CD21^+^ B cells

Peripheral blood mononuclear cells (PBMCs) were isolated from the blood of healthy neonatal piglets (5–7-day-old), weaned piglets (5–6-week-old), and gilts (6–7-month-old) by density centrifugation using a porcine peripheral blood lymphocyte separation kit (Solarbio Science & Technology, Beijing, China). The pigs were purchased from SPF Pig Breeding Management Center, Beijing, China and were double negative for PEDV, transmissible gastroenteritis virus (TGEV), porcine reproductive and respiratory virus (PRRSV), and porcine circovirus type 2 (PCV2) nucleic acid and their antibodies. The blood samples from pigs were diluted at a 1:1 ratio in peripheral blood lymphocyte separation and centrifuged at 2,000 rpm for 20 min. The white layer was collected and washed with phosphate-buffered saline (PBS) at 2,000 rpm for 10 min; then the supernatant was completely removed. The cells were resuspended and labeled with 20 μl FITC-CD21 antibody at 4°C for 15 min. Then, PBMCs were washed with 1 ml buffer and incubated with 10 μl anti-FITC MicroBeads (Miltenyi Biotech, Bergisch Gladbach, Germany) at 4°C for 20 min. Finally, the cells were washed with 2 ml buffer and resuspended in up to 500 μl buffer. The MS column was placed in the magnetic field of the magnet-activated cell sorting (MACS) separator. The column was prepared by rinsing with 1 ml buffer. The cells were added to the column. Magnetically labeled cells were flushed out by firmly pushing the plunger into the tube, and CD21^+^ B cells were harvested. The purification of CD21^+^ B cells was more than 90%, which could be used in subsequent experiments.

### Flow cytometric analysis

CD21^+^B cells sorted from neonatal piglets, weaned piglets, and gilts were seeded in 24-well culture plates at 2 × 10^5^ cells/well and then infected with PEDV virulent (QY2016) and attenuated strains (CV777) at a multiplicity of infection (MOI) of 0.2 for 24 h at 37°C, respectively. At the same time, a negative control group was designed and treated with RPMI-1640. The cells were collected and stained with the indicated antibody. After surface staining, the cells were washed with PBS for three times. Then, the cell counts were performed using a BD flow cytometer and phenotypically analyzed by BD FACS software (BD Biosciences).

### ELISA

CD21^+^B cells sorted from neonatal piglets, nursery piglets, and gilts were seeded in 96-well culture plates at 5 × 10^4^ cells/well and then infected with PEDV virulent (QY2016) and attenuated strains (CV777) at an MOI of 0.5 at 37°C. At 0, 24, and 48 h post infection (hpi), the supernatants were collected for cytokine detection using the ELISA kit. In addition, the supernatants were collected for antibody detection using the PEDV-IgG/IgM/IgA ELISA kit at 0, 4, and 7 days post infection (dpi). Detailed steps are as follows. Diluted standards and 100 μl supernatants from each pig were added to the assay plate, which was incubated at 37°C for 1 h. After washing the plate with wash buffer five times, horseradish peroxidase (HRP)-conjugated antibodies were added to each well. The plate was incubated at 37°C for 30 min, and each well was washed five times. Subsequently, tetramethylbenzidine (TMB) solution was added to each well to incubate in the dark at room temperature for 15 min. Finally, a stop solution was added, and the optical density (OD) of each mixture was read at 450 nm within 15 min.

### Statistical analysis

All experiments were performed independently three times, and variables are expressed as the means ± standard deviation (SD). Statistical analyses were performed using a *t-*test. The *p*-value of < 0.05 was considered significant, the *p*-value of < 0.01 was considered very significant, and the *p*-value of < 0.001 was considered extremely significant.

## Results

### The expression of surface molecules on B cells

**Figure 1:**
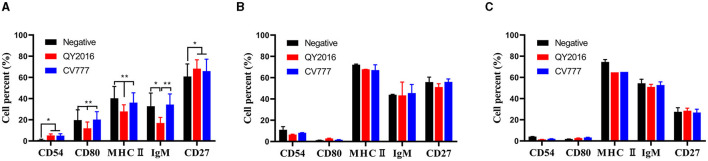
Effects of PEDV on B cell surface molecules. The percentage of CD54, CD80, class II MHC molecules, IgM, and CD27 on the surface of B cells from neonate piglets **(A)**, nursery piglets **(B)**, and gilts **(C)** after PEDV virulent (QY2016) and attenuated strains (CV777) infection at 24 hpi were detected by flow cytometry. * or ** indicate significant differences between two groups statistically (*p* < 0.05 and *p* < 0.001).

We investigated the effect of PEDV virulent and attenuated strains on the expression of CD54, CD80, class II MHC molecules, IgM, and CD27 on the surface of B cells from neonate piglets, nursery piglets, and gilts. As shown in Figure 1A, compared with the PEDV-CV777 strains infection group and the negative group, the percentage of CD80, MHC II, and IgM expressed on the surface of B cells from neonatal piglets was significantly decreased after thePEDV-QY2016 strains infection at 24 hpi. It was found that the percentage of CD54 and CD27 expressed on the surface of B cells was significantly higher in the PEDV-QY2016 or PEDV-CV777 strains infection group than those in the negative group. However, compared with the negative group, the percentages of CD54, CD80, MHC II, IgM, and CD27 on the surface of B cells from nursery piglets and gilts in the PEDV-infected group were not significantly different (Figures 1B, C). These results showed that PEDV mainly affects the expression of B cell surface molecules in neonate piglets.

### The levels of cytokines secreted by B cells

**Figure 2:**
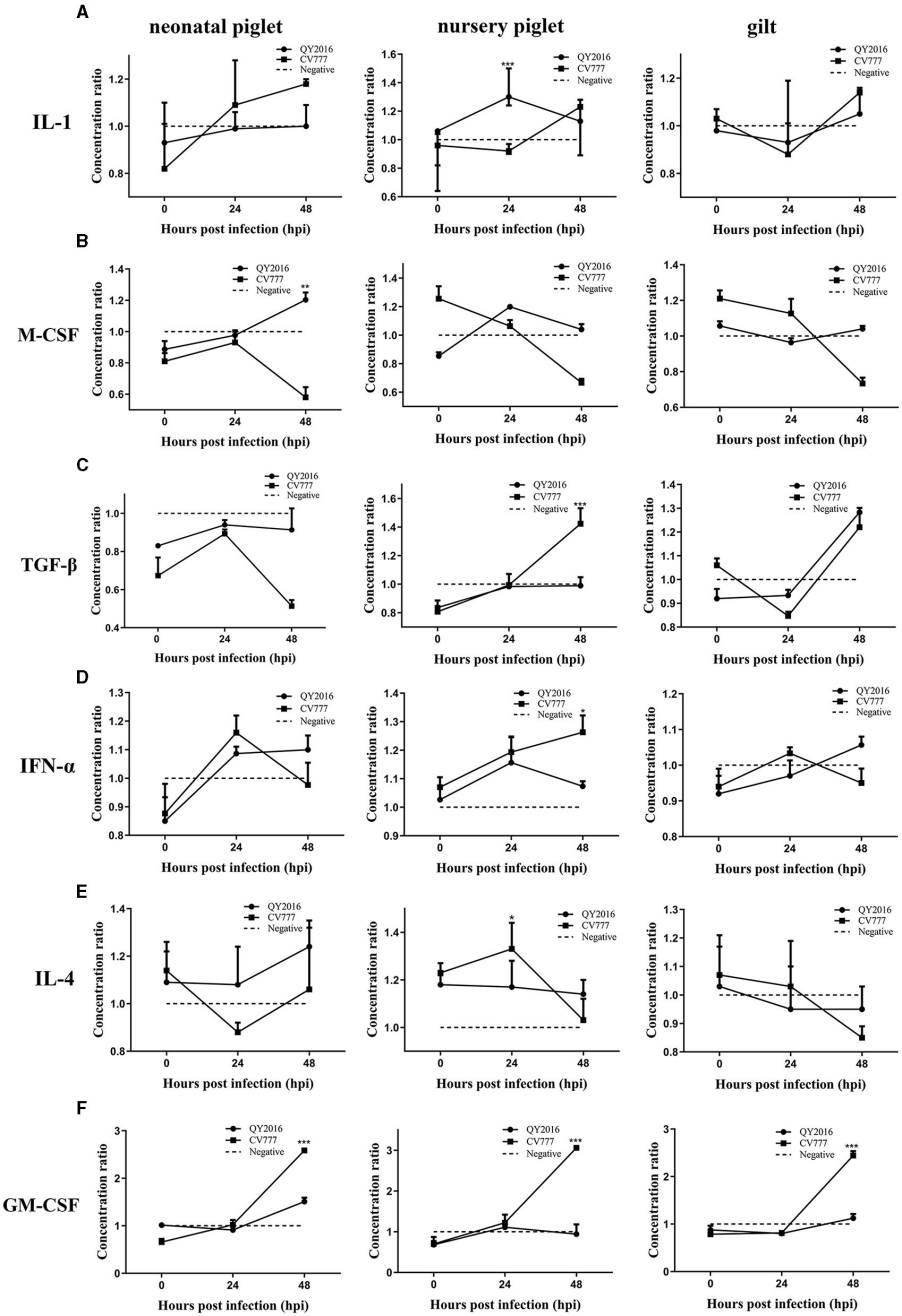
Dynamic changes of IL-1, M-CSF, TGF-β, IFN-α, IL-4, and GM-CSF secreted by B cells infected with PEDV. The supernatant of PEDV-infected B cells from neonate piglets, nursery piglets, and gilts was collected after PEDV virulent (QY2016) and attenuated strains (CV777) infection at 0, 24, and 48 hpi, and the secretion level of cytokine IL-1 **(A)**, M-CSF **(B)**, TGF-β **(C)**, IFN-α **(D)**, IL-4 **(E)**, and GM-CSF **(F)** was detected by ELISA. The concentration ratio is the result of the PEDV infection group compared with the negative control group. *Indicates significant difference between PEDV virulent (QY2016) and attenuated strains (CV777; *p* < 0.05). **Indicates significant difference between PEDV virulent (QY2016) and attenuated strains (CV777; *p* < 0.01). ***Indicates significant difference between PEDV virulent (QY2016) and attenuated strains (CV777; *p* < 0.001).

We next examined the differences in the secretion of some important cytokines in B cells during PEDV infection. Compared with the negative group, the PEDV-QY2016 strains infection group had significantly increased the synthesis of interleukin (IL)-1 and macrophage colony-stimulating factor (M-CSF), that is, the IL-1 levels were significantly upregulated in nursery piglets at 24 hpi (Figure 2A), and the M-CSF significantly increased in neonatal piglets at 48 hpi (Figure 2B). In addition, after infection with PEDV-CV777 strains, it was found that the secretion levels of transforming growth factor (TGF)-β and IFN-α were significantly higher in B cells from nursery piglets at 48 hpi (Figures 2C, D) and those of IL-4 significantly increased from nursery piglets at 24 hpi (Figure 2E). Notably, PEDV-CV777 strains had significantly increased granulocyte-macrophage colony-stimulating factor (GM-CSF) secretion in B cells from neonatal piglets, nursery piglets, and gilts at 48 hpi (Figure 2F). These results showed that the cytokine profiles secreted by pig's B cells were different under the stimulation of PEDV-QY2016 and PEDV-CV777 strains, and the cytokine responses of B cells from newborn piglets, nursery pigs, and gilts were varying under the same strain.

### The change of antibody secreted by B cells

**Figure 3:**
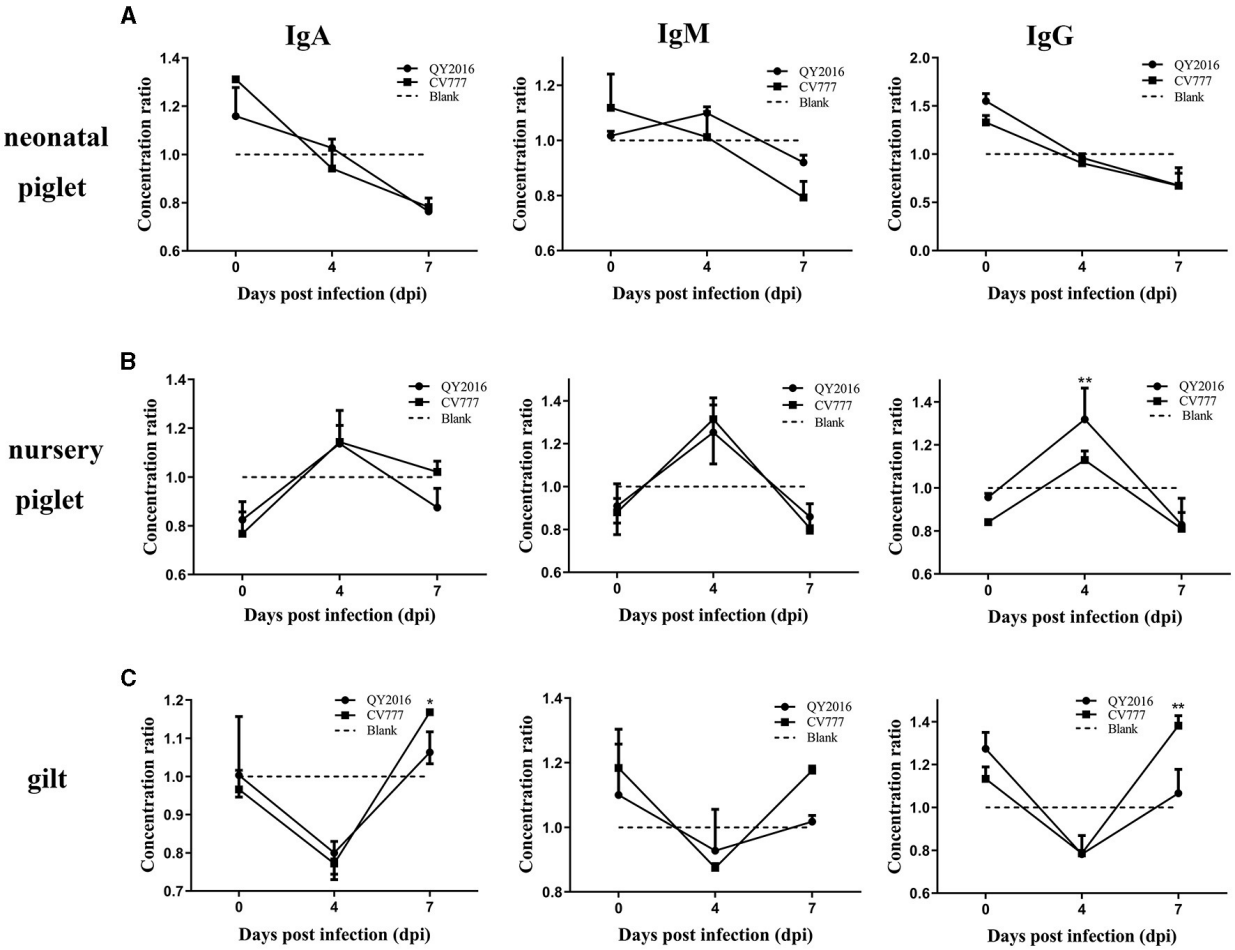
Dynamic changes of IgA, IgM, and IgG secreted by PEDV-infected B cells from neonate piglets, nursery piglets, and gilts. The supernatant of PEDV-infected B cells from neonate piglets **(A)**, nursery piglets **(B)**, and gilts **(C)** was collected after PEDV virulent (QY2016) and attenuated strains (CV777) infection at 0, 4, and 7 dpi, and the level of IgA, IgM, and IgG was detected by ELISA. The concentration ratio is the concentration of IgA, IgM, or IgG in the PEDV-infected group vs. the negative control group. *Indicates significant difference between PEDV virulent (QY2016) and attenuated strains (CV777; *p* < 0.05). **Indicates significant difference between PEDV virulent (QY2016) and attenuated strains (CV777; *p* < 0.01).

In testing for antibodies secreted by B cells in this study, no difference was found in the levels of IgA, IgM, and IgG secreted by B cells between the PEDV-QY2016 and PEDV-CV777 strains infection group, remaining relatively consistent on a downward trend in the neonatal piglet B cells from 4 to 7 dpi (Figure 3A). In contrast, we observed that PEDV-QY2016 and PEDV-CV777 could induce the secretion of IgA, IgM, and IgG in B cells from nursery piglets at 4 dpi, while the level of IgG was significantly higher in the PEDV-QY2016 strains infection group than that in the PEDV-CV777 strains infection group in nursery piglets at 4 dpi. However, the levels of IgA and IgM showed no significant difference in the PEDV-QY2016 and PEDV-CV777 strains infection groups in nursery piglets from 4 to 7 dpi (Figure 3B). Moreover, the IgA, IgM, and IgG secreted by B cells between the PEDV-QY2016 and PEDV-CV777 strains infection groups maintained a relatively consistent upward trend in the gilt B cells from 4 to 7 dpi. The secretion of IgA, IgM, and IgG in gilts B cells infected with the PEDV-CV777 strains as significantly higher than that observed in gilts B cells infected with the PEDV-QY2016 strains at 7 dpi (Figure 3C). These results indicated that both PEDV-QY2016 or PEDV-CV777 strains could induce the secretion of IgA, IgM, or IgG in B cells of nursery piglets and gilts, but could not induce the secretion of IgA, IgM, and IgG in B cells of neonatal piglets.

## Discussion

PEDV is a highly contagious enterovirus that could infect pigs of all ages, causing serious harm to the pig industry worldwide (Jung et al., 2020). The PEDV infection is controlled by a complex and concerted response of host innate immunity and adaptive immunity (Lin et al., 2022; Zhang et al., 2023). Thorough knowledge of the humoral immune response to PEDV is essential to better understand host defense mechanisms. B cells play an important role in the humoral immune response (Lipsky, 2001). However, little is known about the role of B cells in humoral immune responses to PEDV. Therefore, in this study, pigs of different ages were selected as research objects to study the surface molecules, cytokine secretion, and antibody production of B cells stimulated by PEDV virulent (QY2016) and attenuated strains (CV777), clarifying the effects of different strains on B-cell response and the difference of age on B-cell immune response induced by PEDV. Indeed, antiviral immunity-induced complex innate and adaptive antiviral immune responses require the activation of many immune cells, including T cells, natural killer cells, macrophages, and B cells. However, because experiments with animals, especially larger animals, are more expensive or limited by animal ethics, many experiments are often performed *in vitro* using cell or tissue models (Olayanju et al., 2019; Zhang et al., 2022). Therefore, although this study utilized B cells as an *in vitro* model, it does not fully recapitulate the complexity of anti-infection responses in hosts. The results can still reflect the immune response characteristics of porcine B cells stimulated by different PEDV strains and provide an important basis for further studies *in vivo*.

B cells are derived from mammalian bone marrow, and peripheral blood lymphocytes account for 5%−25% of the total B cells (Chousterman and Swirski, 2015; Wang et al., 2020). In addition, B cells have the characteristics of convenient source and avoidance of contamination. Therefore, based on various studies (Crawley et al., 2003), CD21^+^ B cells were selected as the experimental model to analyze the immune responses of B cells from pigs of different ages against PEDV virulent or attenuated strains. The costimulatory molecule expressions of B cells are closely related to B cell antigen presentation, B cell activation, and proliferation (Cai and Yin, 2020). For example, activated B cells could upregulate the expression of MHC II, and antigen peptides can be combined with MHC II to form complexes and present them to T cells (Sproul et al., 2000). The CD80 molecule expressed on activated B cells is believed to be important in eliciting a T cell response via its ligands CD28 and CTLA-4 (Linsley et al., 1991; Azuma et al., 1993; Freeman et al., 1993). The CD54, also known as intercellular adhesion molecule-1 (ICAM-1), is involved in the activation process of T cells and B cells and plays a role in inflammatory response and acquired immune response. In this study, compared with the negative controls, the percentages of CD54, CD80, MHC II, IgM, and CD27 on the surface of PEDV-infected B cells from neonatal piglets were significantly different from those of nursery piglets and gilts. Based on B cell phenotype detection, the results indicated that age affected the immune response of B cells activated by PEDV virulent (QY2016) and attenuated strains (CV777), that is, PEDV had weak activation ability on B cells from nursery piglets and gilts.

B cells could regulate the immune response by secreting cytokines. B cells could produce a variety of inflammatory cytokines, such as IL-1, IL-6, and IL-10, which are involved in antiviral defense, and these cytokines not only act as innate signals but also affect T-cell polarization (Shen and Fillatreau, 2015). At the same time, the division and differentiation of B cells into immunoglobulin-producing plasma cells is induced by IL-4(Lam et al., 2020). Moreover, B cells could also produce TGF-β, which induces naive CD4^+^ T cells and immature DCs to differentiate into Treg cells and tolerate DC transformation, respectively (Matsushita, 2019). The GM-CSF is a potent activator of various cells such as granulocytes, monocytes/macrophages, and eosinophils (Hamilton and Achuthan, 2013). The present study found that the inflammatory, regulatory, and other cytokines secreted by pigs of different ages significantly differed after PEDV virulent (QY2016) and attenuated strains (CV777) infection.

In addition to antigen presentation and cytokine secretion, B cells play a critical role in the process of anti-PEDV antibody production. Clinically, to control PEDV infection, gilt herds are immunized using the live and attenuated PEDV vaccines to stimulate antibodies against PEDV via the gut-mammary axis. The antibodies developed against PEDV in gilts could be transferred to neonatal piglets via colostrum/milk (Langel et al., 2016). Meanwhile, the number of studies have suggested that antibodies induced by PEDV play a role in protecting piglets from PEDV infection (Langel et al., 2019; Wen et al., 2019). Recently, we have reported that 21-day-old piglets have poor systemic and intestinal isotype-specific antibody responses and weak serum-neutralizing activities against the PEDV strain (Guo et al., 2020). However, several studies have found that high PEDV-specific IgG and IgA were produced after gilt infection (Gerber et al., 2014; Ouyang et al., 2015). Similarly, we found that neither the PEDV virulent (QY2016) nor the attenuated strains (CV777) could induce the secretion of IgA, IgM, and IgG of B cells from neonatal piglets, while both strains could induce the secretion of IgA, IgM, and IgG of B cells from nursery piglets and gilts. The secretion of IgA, IgM, and IgG was significantly higher in gilts B cells infected with the PEDV-CV777 strains than that in gilts B cells infected with the PEDV-QY2016 strains. It was suggested that the B-cell antibody response induced by PEDV was closely related to the ages of pigs and the virulence of the PEDV strain. In addition, this study provides an important reference for explaining why PEDV mainly causes the disease in neonatal piglets and suggests that the PEDV attenuated strain is not suitable for the immunization of neonatal piglets.

For the first time, this study evaluates the immune response of B cells from pigs of different ages to PEDV virulent and attenuated strains *in vitro*. The results indicate that the surface molecule expression, cytokine secretion, and antibody production of B cells induced by PEDV are closely related to the ages of pigs and the virulence of the PEDV strain. Compared with the PEDV-CV777 strains, B cell responses to PEDV-QY2016 strains are weaker, and the immune response of B cells of neonatal piglets and gilts to PEDV is lower than that of nursery piglets. This study is of great significance to elucidate the pathogenic mechanism of PEDV and analyze the characteristics of response to PEDV in pigs of different ages.

## Data availability statement

The original contributions presented in the study are included in the article/supplementary material, further inquiries can be directed to the corresponding author.

## Ethics statement

The animal study was approved by Hebei Agricultural University (China) Animal Welfare and Ethical Review Board (Permit Number: 1820026). The study was conducted in accordance with the local legislation and institutional requirements.

## Author contributions

CY: Data curation, Project administration, Writing—original draft. XZ: Data curation, Project administration, Writing—original draft. YF: Data curation, Writing—review & editing. LC: Project administration, Writing—review & editing. YL: Project administration, Writing—review & editing. TL: Resources, Writing—review & editing. QS: Funding acquisition, Supervision, Writing—review & editing.
